# Low-pathogenicity mycoplasma species induce immunoparesis and are highly prevalent amongst patients with ventilator-associated pneumonia

**DOI:** 10.1186/cc14169

**Published:** 2015-03-16

**Authors:** TJ Nolan, N Gadsby, TP Hellyer, K Templeton, R McMullan, J McKenna, J Rennie, CT Robb, TS Walsh, AG Rossi, AJ Simpson, A Conway Morris

**Affiliations:** 1 University of Edinburgh, UK; 2 NHS Lothian, Edinburgh, UK; 3 Newcastle University, Newcastle, UK; 4 Queen's University, Belfast, UK; 5 Belfast Health and Social Care Trust, Belfast, UK; 6 University of Cambridge, UK

## Introduction

Ventilator-associated pneumonia (VAP) remains a significant problem within ICUs. There is a growing recognition of the impact of critical-illness-induced immunoparesis on the pathogenesis of VAP, but the mechanisms of this immunoparesis remain incompletely understood. We hypothesised that, because of limitations in their routine detection, *Mycoplasmataceae *are more prevalent amongst patients with VAP than previously recognised, and that these organisms potentially impair immune cell function.

## Methods

Two cohorts [1,2], totalling 159 patients, were recruited from 12 UK ICUs; all patients had suspected VAP and underwent bronchoscopy and bronchoalveolar lavage. VAP was defined as growth of organisms at >10^4 ^CFU/ml on conventional culture. Thirty-six healthy donors underwent lavage for comparison. Samples were tested for *Mycoplasmataceae *(*Mycoplasma *and *Ureaplasma *spp.) by PCR, and positive samples underwent sequencing for speciation. Additionally, healthy donor monocytes and macrophages (MDM) were exposed to *Mycoplasma salivarium *and their ability to respond to lipopolysaccharide and undertake phagocytosis was assessed.

## Results

*Mycoplasmataceae *were found in 48% of patients with VAP, compared with 14% of patients without VAP (*P *< 0.0001). Patients with sterile lavage had a similar prevalence to healthy donor lavage (10 vs. 8%, *P *= 0.54). The commonest organism identified was *M. salivarium*. Human blood monocytes and MDM incubated with *M. salivarium *displayed impaired cytokine responses to lipopolysaccharide and MDM demonstrated impaired phagocytosis.

## Conclusion

This study demonstrates a high prevalence of *Mycoplasmataceae *amongst patients with VAP, with a markedly lower prevalence amongst patients with suspected VAP in whom subsequent cultures refuted the diagnosis. The commonest organism found, *M. salivarium*, is able to profoundly impair the functions of key immune cells and thus suggests that *Mycoplasmataceae *may contribute to VAP pathogenesis.
